# Cognitive impairment indicator for the neuropsychological test batteries in the Canadian Longitudinal Study on Aging: definition and evidence for validity

**DOI:** 10.1186/s13195-023-01317-3

**Published:** 2023-10-05

**Authors:** Megan E. O’Connell, Helena Kadlec, Lauren E. Griffith, Christina Wolfson, Geva Maimon, Vanessa Taler, Susan Kirkland, Parminder Raina

**Affiliations:** 1 Department of Psychology and Health Studies, University of Saskatchewan, 9 Campus Drive, Arts 182, Saskatoon, SK S7N 5A5 Canada; 2 Institute On Aging & Lifelong Health, University of Victoria, STN CSC, PO Box 1700, Victoria, BC V8W 2Y2 Canada; 3 Department of Health Research Methods, Evidence, and Impact, McMaster University, 175 Longwood Rd. S. Suite 309a, Hamilton, ON L8P 0A1 Canada; 4 Department of Epidemiology and Biostatistics and Occupational Health, School of Population and Global Health, McGill University, 2001 McGill College Avenue Suite 1200, Montreal, QC H3A 1G1 Canada; 5 CLSA Data Curation Centre, Research Institute of the McGill University Health Centre, 2155 Guy Street, 4th Floor, Montreal, QC H3H 2R9 Canada; 6 School of Psychology, University of Ottawa, 136 Jean Jacques Lussier, Vanier Hall, Ottawa, ON K1N 6N5 Canada; 7 Department of Community Health and Epidemiology, Dalhousie University, 5790 University Ave, Halifax, NS B3H 1V7 Canada; 8 Department of Health Research Methods, Evidence and Impact, Faculty of Health Sciences, McMaster Institute for Research On Aging & Labarge Centre for Mobility in Aging, McMaster University, MIP Suite 309A, 1280 Main St. W, Hamilton, ON L8S 4K1 Canada

**Keywords:** Baserates, Medical conditions, Normative data, Measurement, Cognitive impairment indicator, Neuropsychological battery, CLSA

## Abstract

**Background:**

Prevalence of overall cognitive impairment based on each participant’s performance across a neuropsychological battery is challenging; consequently, we define and validate a dichotomous cognitive impairment/no cognitive indicator (CII) using a neuropsychological battery administered in a population-based study. This CII approximates the clinical practice of interpretation across a neuropsychological battery and can be applied to any neuropsychological dataset.

**Methods:**

Using data from participants aged 45–85 in the Canadian Longitudinal Study on Aging receiving a telephone-administered neuropsychological battery (Tracking, *N* = 21,241) or a longer in-person battery (Comprehensive, *N* = 30,097), impairment was determined for each neuropsychological test based on comparison with normative data. We adjusted for the joint probability of abnormally low scores on multiple neuropsychological tests using baserates of low scores demonstrated in the normative samples and created a dichotomous CII (i.e., cognitive impairment vs no cognitive impairment). Convergent and discriminant validity of the CII were assessed with logistic regression analyses.

**Results:**

Using the CII, the prevalence of cognitive impairment was 4.3% in the Tracking and 5.0% in the Comprehensive cohorts. The CII demonstrated strong convergent and discriminant validity.

**Conclusions:**

The approach for the CII is a feasible method to identify participants who demonstrate cognitive impairment on a battery of tests. These methods can be applied in other epidemiological studies that use neuropsychological batteries.

The Canadian Longitudinal Study on Aging is a population-based study of adults aged 45–85 at study entry [1, 2]. A major strength of the CLSA is the use of neuropsychological batteries to measure cognition. Unlike cognitive screening tests, neuropsychological batteries do not provide overall summaries of cognitive status and cannot be easily combined. Other longitudinal studies [3] have used clinician assessments to determine evidence for overall cognitive impairment from participants’ performance on a neuropsychological battery. Cut-offs for composite scores created from neuropsychological batteries have been developed for the Health and Retirement Study [4, 5] including a recent algorithm using machine learning approaches [6], but these approaches were trained on a subsample who received a clinician diagnosis in the Aging, Demographic, and Memory Study [7]. A clinician assessment was not undertaken in the CLSA. We present an approach to determine cognitive impairment based on participant performance across the CLSA neuropsychological battery and present evidence for its validity.

Determination of abnormally low performance on each neuropsychological test relies on comparison with a normative sample and setting a cut-off for impairment. Determining a person’s overall cognitive impairment based on performance across multiple tests in a neuropsychological battery is complex. A person can perform within normal limits or perform well below average (i.e., abnormally low performance) on any number of the neuropsychological tests. If a person obtains some abnormally low test scores, does this reflect overall cognitive impairment? If the cut-off for abnormally low scores is the 5th percentile relative to a comparison with normative data, every person has a 5% chance of their performance on a given test being recorded as abnormally low, even in the absence of true cognitive impairment. With several tests in a battery, the probability of at least one test falling below the 5th percentile in the absence of cognitive impairment is greater than 5%. The greater the number of tests in the battery, the higher the probability of a false conclusion of ‘impairment’ [8–10]. Ignoring this inflated probability of spuriously impaired scores results in an overestimation of cognitive impairment [8, 11]. Correcting for this inflated probability of impaired scores across a battery of neuropsychological tests when making a determination of overall cognitive impairment is imperative to good clinical neuropsychological practice and can be applied using a baserate approach for research, such as with the CLSA and other epidemiological studies. A baserate approach involves algorithms that estimate the expected number of very low scores [12], facilitating the interpretation of a neuropsychological battery [13]. The baserate approach to determine spurious low scores using Crawford et al.’s [12] algorithm has evidence for its validity [9, 10].

Normative comparison standards for the CLSA were created for each of the four neuropsychological tests used in the Tracking cohort [14] and for seven of the eight tests used in the Comprehensive cohort.

The CII is derived from the participant’s performance on each test in the battery, where each test score is compared with normative data from the CLSA [14]. Crawford and colleagues’ [12] baserate algorithm was used to adjust for the probability of spuriously low scores before classifying the person’s overall performance. To assess the convergent and discriminant validity of the CII, we used the participants’ responses to a series of questions about physician-diagnosed chronic conditions. Based on the literature, we had three sets of hypotheses. We hypothesized (1) that the CII would be associated with neurological conditions that can cause cognitive impairment such as dementia or Alzheimer’s disease [15], stroke [16], transient ischemic attack [17], multiple sclerosis [18], Parkinson’s disease [19], and epilepsy [20]; (2) that the CII would be strongly associated with physician-diagnosed memory problems; (3) that medical conditions that are risk factors for cognitive impairment would be associated with the CII, but to a lesser degree. The chronic conditions that are risk factors for cognitive impairment include diabetes [21], hypertension [22], cardiac diseases [23], major depressive disorder [24], peripheral vascular disease [25], kidney disease [26], and thyroid dysfunction [27]. We did not expect the CII to be associated with allergies [28], arthritis [29], migraines [30], osteoporosis [31], history of cancer [32], ulcers, or back pain. We had no a priori hypotheses for bowel or urinary incontinence because these are features of advanced neurological conditions, and the cognitive consequences for these as stand-alone conditions are not well-studied.

## Methods

### Aim, design, and setting

The aim is to develop and validate a cognitive impairment indicator that summarizes cognitive performance across a neuropsychological battery. Prospective cohort design, but cross-sectional analyses for the current project. The setting is community-based.

### Participants

CLSA participants have been described elsewhere [1]. Briefly, an age-stratified random sample from the Canadian population between the age of 45 and 85 years was selected for the Tracking cohort, and random samples of participants residing near one of eleven data collection sites across Canada were selected for the Comprehensive cohort. The Tracking cohort (*N* = 21,241) was administered questionnaires over the telephone; including yes/no questions about having been diagnosed by a physician as having a chronic condition (34 conditions), four neuropsychological tests (see [33] for a description of the data collection protocol and tools). Participants in the Comprehensive cohort (*N* = 30,097) were assessed in. The descriptions of the two cohorts are shown in Table 1. Although the CLSA is an ongoing longitudinal study, the data for the current project were cross-sectional from the baseline data collection phase.

**Table 1:** Description of the Tracking and Comprehensive cohorts of the CLSA at baseline

**Demographic variables and sample sizes for cognitive tests**	**Tracking **(*N* = 21,241)	**Comprehensive **(*N* = 30,097)
Age (years): mean (SD)	63.01 (10.67)	62.96 (10.25)
Sex: number (%) men	10,406 (49.0%)	14,777 (49.1%)
Education level: number (%)		
< high school	1986 (9.4%)	1643 (5.5%)
High school graduate	2880 (13.6%)	2839 (9.4%)
Some post-secondary	1620 (7.6%)	2238 (7.4%)
Post-secondary degree/diploma	14,661 (69.1%)	23,327 (77.5%)
(Missing)	(94)	(50)
REY I immediate recall: number (%)		
English	15,989 (81.7%)	23,456 (80.7%)
French	3587 (17.6%)	5615 (19.3%)
(Missing or inconsistent)	(1665)	(1026)
REY II delayed recall: number (%)		
English	16,038 (82.4%)	23,217 (80.7%)
French	3419 (17.6%)	5542 (19.3%)
(Missing or inconsistent)	(1784)	(1338)
MAT: number (%)		
English	15,781 (86.2%)	22,575 (80.5%)
French	2534 (13.8%)	5480 (19.5%)
(Missing or inconsistent language used)	(2926)	(2042)
Animal Fluency: number (%)		
English	16,783 (85.9%)	23,550 (81.6%)
French	2762 (14.1%)	5300 (18.4%)
(Bilingual, missing or inconsistent language used)	(1696)	(1247)
Stroop Interference		
English	n/a	24,323 (80.8%)
French		5746 (19.1%)
(Missing)		(28)
FAS total		
English	n/a	22,886 (82.4%)
French		4905 (17.6%)
Missing or inconsistent language used		(2,306)

*CLSA* Canadian Longitudinal Study on Aging, *SD* standard deviation, *REY I* Rey Auditory Verbal Learning Test immediate recall, *REY II* Rey Auditory Verbal Learning Test 5-min delayed recall, *MAT* Mental Alternation Test, *FAS* Letters from the Controlled Oral Word Association Test, *Stroop Interference* Victoria Stroop Dots card/Colours card times

### Measures

#### Neuropsychological tests

The neuropsychological tests used in CLSA are described in more detail elsewhere [34, 35], but included the following tests administered by telephone to the Tracking cohort: Rey Auditory Verbal Learning Test immediate recall (REY I) and 5-min delayed recall (REY II), the Mental Alternation Test (MAT), and Animal Fluency (AF; we used AF2 scores that are consistent with scoring rules used clinically [14]).

The Comprehensive Cohort completed testing in-person, including the above four tests, as well as the Controlled Oral Word Association Test (total score was used for the letters FAS) and a modified version of the Victoria Stroop Test (Stroop Interference—interference ratio time it took to complete the task on the “color” task divided by performance on the “dot” task). For all but the Stroop test, higher scores indicated better cognitive performance; for the Stroop Interference score, lower scores indicated less interference. Summaries of the raw performances on these tests (i.e., not normed) for the whole sample in both cohorts are shown in the top of Table 2. To create normatively corrected scores Stroop Interference scores were reversed, making interpretation of these scores consistent with the other neuropsychological tests, and for all analyses the norm-corrected Stroop scores were used. Stroop errors were not used for the analyses due to the extreme skewness in this variable (i.e., most participants did not make errors). The Comprehensive cohort also received a choice reaction time and two prospective memory tasks, but these tests were not used in the current study due to problems in administration or highly skewed distributions.

**Table 2:** Performance on neuropsychological battery

Neuropsychological test	Tracking			Comprehensive		
** “Raw” test scores**	*N*	Mean	*SD*	*N*	Mean	*SD*
REY I immediate recall	19,576	5.91	2.35	29,073	5.85	1.91
REY II delayed recall	19,462	4.36	2.59	29,041	4.04	2.17
Animal Fluency	20,432	20.97	6.52	29,365	21.41	6.47
MAT	18,823	25.98	9.60	28,606	26.54	8.75
Stroop Interference	-	-	-	29,675	2.159	0.731
FAS total	-	-	-	28,994	39.21	12.79
**Cognitive impairment on individual tests**	**Available *N***	**# Imp**	**%**^**a**^	**Available *N***	**# Imp**	**%**^**a**^
Based on REY I immediate recall	19,488	1176	5.5	29,024	1671	5.8
Based on REY II delayed recall	19,374	1026	5.3	28,715	1560	5.4
Based on Animal Fluency	19,472	1100	5.6	28,804	1521	5.3
Based on MAT score	18,246	1654	7.8	28,014	2122	7.6
Based on Stroop Interference	-	-	-	29,626	1602	5.4
Based on FAS total	-	-	-	27,724	1512	5.5
**Cognitive impairment on overall 4-test battery **^**b**^						
Cognitively Impaired	16,371	664	3.1	27,203	983	3.6
Not impaired		15,707	95.9		26,220	96.4
Unclassified due to missing data — out of total *N*		(4870)	(22.9)		(2894)	(9.6)
**Cognitive impairment on overall 6-test battery** ^c^						
Cognitively impaired	-	-	-	25,168	1528	6.1
Not impaired					23,640	93.9
(Unclassified due to missing data — out of total *N*)					(4929)	(16.4)

*SD* standard deviation, *REY I* Rey Auditory Verbal Learning Test immediate recall, *REY II* Rey Auditory Verbal Learning Test 5-min delayed recall, *AF2* Animal Fluency, *MAT* Mental Alternation Test, *Stroop Interference* Victoria Stroop Dots card/Colours card times, *FAS* Letters from the Controlled Oral Word Association Test

^a^% is the valid percent excluding missing values. Missing values Tracking raw scores for REY I 8.3%; REY II 8.8%; AF2 8.4%; MAT 14.2%. Missing values for Comprehensive: REY I 3.6%; REY II 4.6%; AF2 4.3%; MAT 6.9%; STP 1.6%; and FAS 7.9%

^b^The 4-test battery consists of REY I, REYII, AF2, and MAT

^c^The 6-test battery consists of REY I, REYII, AF2, MAT, Stroop, and FAS, Comprehensive cohort only

#### Chronic conditions

Participants were asked to respond yes/no to the question: “Has a doctor ever told you that you have (the chronic condition)?” The list of conditions, and the number of participants who responded yes to each, is shown in Table 3. We examined each condition separately for its association with the CII, and we created three groupings based on our a priori hypotheses. One group labeled “Neurological” included participants who reported having a physician diagnosis of dementia or Alzheimer’s disease, memory problems, stroke, transient ischemic attack, multiple sclerosis, parkinsonism or Parkinson’s disease, or epilepsy; versus those who denied any neurological condition. A second group labeled “Risk Neurological” included participants with a self-reported physician diagnosis of at least one known risk factor for cognitive impairment: diabetes, hypertension, cardiac diseases, major depressive disorder, peripheral vascular disease, kidney disease, or thyroid dysfunction versus those reporting none of these conditions. To provide support for the CII with divergent validity, a third group was created with participants who had self-reported conditions for which we did not expect to see an increased likelihood of cognitive impairment: allergies, arthritis, migraines, osteoporosis, history of cancer, ulcers, or back pain. We were unable to create a comparison group of persons who reported none of these conditions because too many participants in each cohort had at least one of these conditions. Consequently, the third group condition was modified to include arthritis, migraines, osteoporosis, history of cancer, or ulcers with the comparison group comprised of those reporting none of these conditions.

**Table 3:** Frequency of chronic conditions by cohort

	**Tracking**		**Comp**	
**Chronic health vondition(s)**^**a**^	***N***	**% **^**b**^	***N***	**% **^**b**^
Osteoarthritis in the knee	3426	16.2	4499	15.1
Osteoarthritis in one or both hips	2087	9.9	2499	8.4
Osteoarthritis in one or both hands	2981	14.1	3857	13
Rheumatoid arthritis	1093	5.2	964	3.2
Asthma	2347	11.1	3984	13.3
Emphysema, chronic bronchitis, chronic obstructive pulmonary disease (COPD), or chronic changes in the lungs due to smoking	1436	6.8	1725	5.8
*Pulmonary — combined category*	*3278*	*15.5*	*5049*	*16.9*
High blood pressure or hypertension	8099	38.1	11,101	37.1
Heart disease (including congestive heart failure, or CHF)	2190	10.3	3503	11.7
Angina (or chest pain due to heart disease)	1148	5.4	1324	4.4
Heart attack or myocardial infarction	1317	6.2	1461	4.9
Peripheral vascular disease or poor circulation in limbs	1518	7.2	1646	5.5
*Cardiovascular — combined category*	*9646*	*45.8*	*13,022*	*44.1*
Stroke	390	1.8	522	1.7
Mini-stroke or TIA (transient ischemic attack)	748	3.5	965	3.2
Memory problem	449	2.1	519	1.7
Dementia or Alzheimer’s disease	43	0.2	68	0.2
Mood disorder	3103	14.6	5144	17.2
Anxiety disorder	1557	7.3	2597	8.7
Parkinsonism or Parkinson’s disease	78	0.4	125	0.4
Multiple sclerosis	141	0.7	202	0.7
Epilepsy	166	0.8	322	1.1
Migraine headaches	2913	13.7	3858	12.9
Intestinal or stomach ulcers	1635	7.7	2275	7.6
Bowel disorder	1836	8.6	2938	9.8
Bowel incontinence	492	2.3	582	1.9
Urinary incontinence	1871	8.8	2516	8.4
Under-active thyroid gland	2444	11.5	3962	13.3
Over-active thyroid gland	466	2.2	724	2.4
Diabetes, borderline diabetes, or high blood sugar	3550	16.7	5310	17.7
Allergies	7849	37.0	11,498	38.6
Osteoporosis	2008	9.5	2689	8.9
Back problems (excl. fibromyalgia & arthritis)	5203	24.5	8384	28
Kidney disease or kidney failure	592	2.8	867	2.9
Cancer	3264	15.4	4637	15.5
Other long-term physical or mental condition	6551	30.8	14,546	48.5
Hearing loss (self-rated as fair or poor)	2661	12.5	3434	11.4
Vision loss (self-rated as fair or poor)	1902	9	2306	7.7

^a^List excludes pneumonia, flu, or other infections in the past year

^b^Percentages are based on the “valid” *N* (i.e., excluding missing values). For TRM, missing values were < 0.9% for individual chronic conditions and < 1.5% for combined categories; for COM, missing values were < 1.3% for individual chronic conditions and 3.9% for combined categories

### Analytic approach

#### Derivation of the cognitive impairment indicator (CII)

For each cohort, the derivation of the CII involved three steps. First, on each neuropsychological test, each participant’s raw score was transformed to a normed score based on comparisons with the neurologically healthy normative sample [14], with regression-based norms correcting for the participant’s age, sex, and education within each language group (referred to hereafter as “normed scores”). In the second step, the participant’s normed score was used to obtain their low score indicator (impaired versus within normal limits) for each neuropsychological test by comparing the participant’s normed score to the cut-off point for abnormally low scores. The cut-off point was the mean from a bootstrapped distribution of scores from the normative sample corresponding to the 5th percentile for each test score. In the third step, the CII was determined for each participant based on her/his performance across the battery of neuropsychological tests. This classification into overall impaired versus non-impaired for the CII incorporated a baserate of low scores. In particular, baserates of the expected proportions of a cognitively healthy population estimated to demonstrate cognitive impairment on any given test were determined using the algorithm created by Crawford and colleagues [12]. The Crawford et al. [12] algorithm uses a Monte Carlo-based method to estimate the probability of obtaining a given number of abnormally low scores. The probability of abnormally low scores increases as the number of tests in the battery increases and is dependent on the test scores’ intercorrelations. This baserate algorithm is based on the intercorrelations of the neuropsychological tests in the cognitively healthy sub-sample (i.e., the normative sample). The likelihood of low scores also depends on the cut-off used, and for the algorithm we selected as the 5th percentile. The algorithm estimates the baserate for the frequency of test scores falling in the abnormally low range that would be expected to occur in a cognitively healthy population.

We used Crawford et al.’s [12] algorithm in our neurologically healthy norming samples to determine how frequently abnormally low scores would occur on the neuropsychological battery of four (Tracking) or six (Comprehensive) intercorrelated normally distributed tests, separately for French- and English-speaking subsamples. Additionally, we completed this for the four tests given to both the Comprehensive cohort and the Tracking cohort to allow for more direct comparisons across the two. Abnormally low scores were defined as equal to or lower than the 5th percentile because these indicate relatively rare outcomes. For the CLSA Tracking cohort, the algorithm by Crawford and colleagues [12] estimated the percentage of a cognitively healthy population presenting with *at least one* abnormally low score on the four-test battery to be 15.9% of the English-speaking and 15.7% of the French-speaking subsamples, which in a clinical setting represents a relatively common outcome. In contrast, only 3.7% of the cognitively healthy population based on the English-speaking subsample and 3.8% of the cognitively healthy population based on the French-speaking subsample were estimated to present with *at least two* abnormally low scores. We propose that the probably of one abnormally low test was too high (over 15%) and the probability of two or more tests was a sufficiently rare baserate likely indicative of cognitive impairment so we used a cutoff of impairment on two or more tests for the CII.

For the Comprehensive cohort, the baserate for at least one abnormally low score on the four-test battery was estimated at 15.6% of the English-speaking and 15.9% for the French-speaking subsample, again a relatively common occurrence, whereas at least two abnormally low scores would be expected to occur with a baserate of 3.5% for both the French- and English-speaking subsamples. We determined that two of the four tests presenting as abnormally low was sufficiently rare to indicate cognitive impairment for the four-test CII in the Comprehensive cohort.

For the six-test Comprehensive battery, the estimated percentage of the population presenting with at least one abnormally low score was 22.6% (22.56% in English and 22.60% in French), whereas 5.8% were estimated to present with two or more low scores (5.81% in English and 5.78% in French) and only 1.4% of the population were estimated to present with three or more abnormally low scores. One abnormally low score was too common (over 22%), but low scores for three or more tests were estimated to occur in less than 2% of the population which was too rare, so we chose to use the cut-off of two or more tests as indicative of cognitive impairment.^1^

In summary, for both Tracking and Comprehensive cohorts, participants who obtained two or more abnormally low test scores, whether in the four-test or the six-test battery, were classified as overall cognitively impaired (CII = 1); otherwise, they were classified as not cognitively impaired (CII = 0). The CII was created for all participants in the CLSA who had complete cognitive data (i.e., four test scores in the Tracking cohort and six test scores in the Comprehensive cohort) and for whom normative comparisons were possible (i.e., they had complete data for age, sex, education level, and language of administration).

#### Concurrent and discriminant validity of the CII

To explore the validity of the CII, we used logistic regression analyses to assess whether individual or groups of chronic medical conditions were associated with CII as posited by our a priori hypotheses (see the “Chronic conditions” section for the groupings). Groups of chronic conditions were created to mitigate concerns about small cell sizes for some of the chronic conditions. In the analyses for groups of chronic conditions, we used sampling weights (version 1.2) [36] that were adjusted for the Canadian population to explore if this impacted the associations with the CII. Sampling weights inflate the observations in the sample to the level of the population to minimize the sampling bias, allowing observations within the sample to be extrapolated to the population of origin.

For the odds ratio (OR) estimates from the logistic regressions, we used the descriptors of magnitude of OR provided by Chen et al. [37], based on a rate of cognitive impairment of 4% in a cognitively healthy group for the 4-test CII: OR = 1.0 to 1.49 as trivial to 1.5 as small, 1.6 to 2.7 as medium, and 2.8 to 5.0 as large (the six-test CII had a higher baserate of cognitive impairment, so OR = 1.5 was classified as small, OR = 2.7 was medium, OR = 4.6 was large). Finally, we calculated the prevalence of cognitive impairment in the Tracking and Comprehensive cohorts with and without sampling weights [36] using the CII based on the same four tests.

## Results

The prevalence of cognitive impairment in the CLSA was 4.3% (4.1% before applying the sampling weights) in the Tracking and 4.3% (3.1% before applying sampling weights) in the Comprehensive cohorts (see Table 2). Table 4 shows the estimated ORs estimates, and their 95% confidence intervals, from the logistic regression analyses for each chronic condition associated with an increased odds of cognitive impairment as indicated by the CII. Dementia or Alzheimer’s disease was associated with a large increased odds of cognitive impairment, and memory problems were associated with medium magnitude OR across both cohorts. Stroke had a medium magnitude OR in the Tracking and a small magnitude OR in the Comprehensive cohort. The OR associated with parkinsonism/Parkinson’s disease and multiple sclerosis was of medium magnitude in Tracking and small magnitude for the 4-test CII Comprehensive, but the OR’s confidence interval included zero when the CII was based on six tests. The Comprehensive cohort received additional health-related questions, including details about traumatic brain injury (TBI) and the location of cancer in the central nervous system (CNS). The CI of the ORs for self-reported residual symptoms from a TBI did not include zero (expB 2.147; CI 1.654–2.787 for the 4 test CII and 1.843; CI 1.472–2.308 for the 6 test CII), but the ORs for cancer of the CNS were not significant likely due to only 36 of the sampling repotting this type of cancer. Bowel incontinence had a medium magnitude OR in the Tracking cohort and in the Comprehensive cohort a small magnitude OR for the 4-test CII and a trivial magnitude OR for the 6-test CII. Peripheral vascular disease, mood disorders, anxiety disorders, epilepsy, and intestinal or stomach ulcers had a small magnitude OR in the Tracking and small to trivial ORs in Comprehensive. Vision impairment had a small magnitude OR and hearing impairment a trivial to small magnitude OR. Some medical conditions presented with statistically significant OR across both cohorts, but the magnitude of the OR was trivial such as for urinary incontinence, diabetes, stomach ulcers, transient ischemic attacks (TIAs), and rheumatoid arthritis. Where remaining conditions were statistically significant inconsistently across cohorts, significant ORs were trivial in magnitude.

**Table 4:** Results of logistic regression with each individual chronic conditions as predictors of overall cognitive impairment^a^

	Tracking^b^		62,		Comprehensive^c^	
Predictor	Odds ratio	95% CI	Odds ratio 4 tests	95% CI 4 tests	Odds ratio 6 tests	95% CI 6 tests
Osteoarthritis	**1.295**	**1.098, 1.528**	.949	.861, 1.046	1.014	.939, 1.094
Rheumatoid arthritis	**1.441**	**1.068, 1.943**	1.172	.834, 1.646	**1.360**	**1.048, 1.766**
Pulmonary related conditions	1.263	1.080, 1.477	1.056	.917, 1.217	1.042	.928, 1.170
High blood pressure or hypertension	**1.205**	**1.031, 1.407**	1.012	.887, 1.155	**1.151**	**1.035, 1.279**
Heart disease (including congestive heart failure, or CHF)	1.158	.907, 1.478	1.251	1.039, 1.507	**1.185**	**1.015, 1.384**
Angina (or chest pain due to heart disease)	1.488	1.106, 2.002	1.294	.977, 1.714	**1.284**	**1.016, 1.623**
Heart attack or myocardial infarction	1.168	.865, 1.578	**1.340**	**1.026, 1.751**	**1.349**	**1.083, 1.680**
Peripheral vascular disease or poor circulation in limbs	**1.776**	**1.388, 2.273**	**1.345**	**1.047, 1.729**	**1.458**	**1.192, 1.784**
Stroke	**3.214**	**2.204, 4.687**	**1.995**	**1.378, 2.890**	**1.951**	**1.425, 2.672**
Mini-stroke or TIA (transient ischemic attack)	**1.456**	**1.016, 2.088**	**1.390**	**1.012, 1.908**	**1.437**	**1.111, 1.859**
Memory problem	**4.373**	**3.233, 5.937**	**3.791**	**2.831, 5.075**	**3.515**	**2.709, 4.561**
Dementia or Alzheimer’s disease	**7.747**	**3.282, 18.286**	**7.226**	**3.703, 14.101**	**7.807**	**4.274, 14.259**
Mood disorder	**1.634**	**1.353, 1.974**	**1.417**	**1.215, 1.651**	**1.299**	**1.143, 1.477**
Anxiety disorder	**1.833**	**1.444, 2.328**	**1.604**	**1.326, 1.941**	**1.272**	**1.074, 1.507**
Parkinsonism or Parkinson’s disease	**3.955**	**1.862, 8.403**	**2.488**	**1.253, 4.941**	1.705	.885, 3.282
Multiple sclerosis	**2.973**	**1.661, 5.323**	**1.976**	**1.095, 3.563**	1.274	.705, 2.300
Epilepsy	1.370	.637, 2.948	1.255	.717, 2.197	**1.582**	**1.045, 2.395**
Migraine headaches	**1.299**	**1.058, 1.596**	1.134	.946, 1.361	1.057	.909, 1.230
Intestinal or stomach ulcers	**1.624**	**1.275, 2.068**	**1.256**	**1.007, 1.568**	1.138	.943, 1.374
Bowel disorder	1.489	1.178, 1.881	1.101	.895, 1.353	1.005	.845, 1.195
Bowel incontinence	**2.886**	**2.069, 4.044**	**1.905**	**1.337, 2.713**	**1.485**	**1.073, 2.054**
Urinary incontinence	**1.441**	**1.136, 1.827**	**1.448**	**1.185, 1.770**	**1.342**	**1.133, 1.589**
Under-active thyroid gland	**1.444**	**1.167, 1.786**	1.055	.876, 1.268	1.092	.941, 1.267
Over-active thyroid gland	**2.342**	**1.606, 3.416**	1.027	.679, 1.551	.900	.631, 1.283
Vision-related conditions	1.003	.878, 1.145	.975	.929,1.025	.995	.954, 1.037
Diabetes, borderline diabetes, or high blood sugar	**1.344**	**1.112, 1.625**	**1.405**	**1.206, 1.636**	**1.551**	**1.373, 1.752**
Allergies	1.014	.892, 1.153	**1.109**	**1.030, 1.194**	**1.140**	**1.075, 1.209**
Osteoporosis	**1.347**	**1.063, 1.707**	.955	.760, 1.201	1.117	.937, 1.332
Back problems (excl. fibromyalgia and arthritis)	.872	.739, 1.029	1.057	.942, 1.187	1.028	.934, 1.132
Kidney disease or kidney failure	1.100	.698, 1.734	1.375	.982, 1.926	1.222	.912, 1.637
Cancer	.962	.776, 1.193	.784	.647, .949	.868	.747, 1.009
Vision impairment	**1.842**	**1.480, 2.291**	**1.893**	**1.572, 2.294**	**1.772**	**1.505, 2.085**
Hearing impairment	**1.444**	**1.176, 1.774**	**1.423**	**1.190, 1.702**	**1.520**	**1.316, 1.755**

^a^Chronic conditions are self-reported by participants in response to questions

**Fig. 1:**
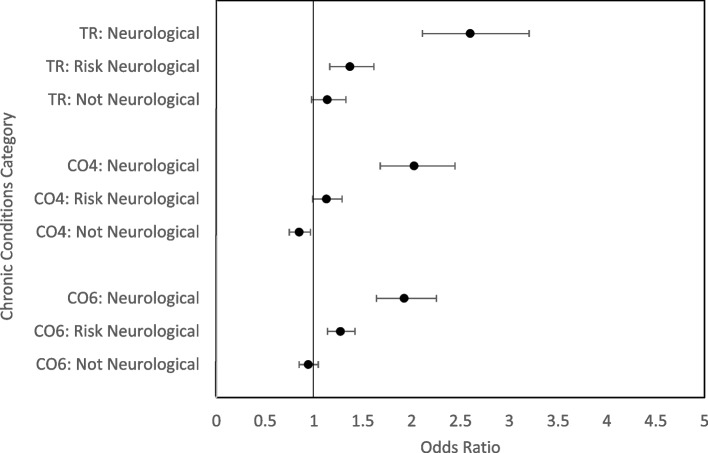
Odds ratios and confidence intervals for Tracking (TR) and Comprehensive (CO) cohorts (4 test CO4 and 6 test CO6 for groups with neurological conditions (neurological), conditions that are risk factors for cognitive impairment (risk neurological), and conditions that we would not expect to be linked to cognition (not neurological)

The grouped variables (see method under *Chronic conditions*) of neurological conditions (*n* = 1694 in Tracking; *n* = 2312 in Comprehensive) presented with a small to medium increased odds of cognitive impairment (see Fig. 1); this finding was expected because these conditions were used as exclusionary criteria for the normative subsample. Figure 1 also shows the trivial magnitude of increased odds of cognitive impairment in conditions that are risk factors for cognitive impairment (*n* = 13,181 in Tracking; *n* = 18,975 in Comprehensive). Finally, there was no evidence of association for cognitive impairment with the grouped variable we did not expect to be associated with the CII (*n* = 11,164 in Tracking; *n* = 15,378 in Comprehensive).

## Discussion

Logistic regression analyses provided evidence for validity of the CII, which suggested the prevalence of cognitive impairment in the CLSA was 4.3% in the Tracking and 4.3% in the Comprehensive cohorts. The approach presented here for identifying cognitive impairment and creating a new CII variable in the CLSA is an approach common in clinical neuropsychology practice [9, 10], but newer in its application to epidemiological aging studies. Understanding the baserates of low scores for neuropsychological tests helps deepen the understanding of neurological conditions such as dementia in epidemiological studies. Kiselica and colleagues [38] studied the Uniform Data Set 3.0 Neuropsychological Battery and found that abnormally low scores were common, and the use of baserates analyses to adjust for the expected number of abnormally low scores displayed in a cognitively healthy subsample helped to predict dementia status. Holdnack and colleagues [39] used baserates of low scores with the National Institutes of Health cognition toolbox and found these agreed well with the reference standard of diagnosed severe traumatic brain injury. Tallying the number of impaired test scores in the neuropsychological battery has been associated with diagnostic criteria for mild cognitive impairment and dementia in the Alzheimer’s Disease Neuroimaging Initiative dataset [40].

Increased odds of cognitive impairment are associated with medical conditions that we would expect to be associated with cognitive impairment, namely diagnosis by a health professional of memory problems, dementia or Alzheimer’s disease, or stroke. This finding was expected because the CII was derived from normed scores and corrected for baserates of low scores expected in the cognitively healthy subsample, which excluded persons with neurological conditions such as dementia, memory problems, and stroke. Although the normative subsample also excluded persons with transient ischemic attacks, Parkinson’s disease (or parkinsonism), multiple sclerosis, and epilepsy, these conditions were not necessarily associated with increased odds of cognitive impairment in the present study. This finding likely reflects the heterogeneity in cognitive status presented by patients diagnosed with these conditions — some but not all individuals patients with these neurological conditions present with cognitive impairment [16].

Diagnosis of mood disorder was associated with a trivial to small increased likelihood of cognitive impairment, consistent with meta-analyses demonstrating a small magnitude of association between depression and cognition [41]. Similarly, sensory loss had a trivial to small association with cognitive impairment, consistent with associations reported between cognition and sensory function [42]. Bowel incontinence was associated with an increased risk of cognitive impairment, potentially due to comorbidities of bowel incontinence with some neurological conditions (e.g., more advanced dementia); alternatively, this finding could be related to a possible link between bowel disorders and cognition via the vagus nerve [43].

In this sample the prevalence of cognitive impairment was relatively low: 4.3% in the Tracking and 5.0% in the Comprehensive cohorts. It is likely that the CLSA sampling procedures led to a low prevalence of cognitive impairment. CLSA participants had to be able to consent without the need for proxy consent procedures at study entry [1], effectively excluding persons with overt cognitive impairment from the study. Nevertheless, the frequency of cognitive impairment in the CLSA appears similar to that reported by Hänninen et al. [44], who identified cognitive impairment (but no dementia) in 5.3% of participants in a population-based study of people aged 60–76. Larrieu and colleagues [45] identified cognitive impairment in 2.8% of a community-based sample. In contrast, other studies have found higher rates of cognitive impairment [46–48]. These varied rates reflect the fact that different ways of conceptualizing cognitive impairment and varied recruitment methods (i.e., were persons with overt cognitive impairment excluded from study entry in the CLSA), which together impact the prevalence in epidemiological studies [49].

### Limitations

The use of self-reported neurological and other medical conditions in this manuscript is a major limitation to these data, and these are the only data available with CLSA at this time. The medical conditions also lacked important details related to cognition. We had insufficient TBI detail to categorize persons as mild, moderate, or severe; no detail on cancer severity, treatment, or how distal the cancer was, we had no detail on the type of MS or whether persons with PD had dementia due to PD. Some of the self-reported conditions, for example, dementia included few people, widening the confidence intervals. Ideally, the validity of the current CII would comprise classification accuracy relative to a clinical evaluation as a gold standard reference. Another limitation of the CII is the limited nature of the tests used none were motor or speed of processing tests, which are the tests we would expect to show deficits for some persons with MS or PD, for example. Another limitation of the CII is the fact that it was only computed if all neuropsychological tests were completed; consequently, the CII was only able to be calculated on 77% of the Tracking cohort, and 83% (for the 6-item) 90% for the 4-item for the Comprehensive cohort due to missing values. The comparability of the findings despite differences in missing values for the different indicators is reassuring, nevertheless, missing data are a limitation and likely reflect missing data from those who have cognitive impairment. Another limitation of the CII is the use of all neuropsychological tests versus only using those that are most sensitive to cognitive impairment, for example, memory tests for Alzheimer’s disease. The chronic conditions reported here would be more or less likely to be associated with impairments on memory or executive function tests, but future work could derive a different CII with tests most sensitive to a condition of interest. In this modified version of the Victoria Stroop Test administered to the comprehensive cohort in CLSA errors were tabulated but not corrected during the task. With this modification, the errors were not captured in the interference score, as it would typically be in the original version of the Victoria Stroop Test. The CII did not include Stroop errors due to extreme skew in this variable and did not include the experimental prospective memory tests that were administered to the Comprehensive cohort in CLSA for this same reason. This CII, therefore, does not use all of the possible neuropsychological tests available in the Comprehensive cohort. The CII is based on one conceptualization of impairment, which was at the 5th percentile. Different conceptualizations of impairment will yield different findings. Finally, the CII is limited by the lack of a premorbid estimate of general cognitive ability, which could be important if the CII were used to help classify persons with suspected cognitive decline.

## Conclusions

The CII appears to have good evidence for convergent and discriminant validity. The baserate approach that is core to the derivation of the CII approximates best practices in clinical neuropsychology, and this approach can be applied to any epidemiological database that includes a battery of neuropsychological tests. It does, however, miss procedural or process approach data, a key aspect of clinical neuropsychology practice that is impossible to detail from summary scores (i.e., raw test scores do not convey how participants approached the task). The CII can be used in future studies using the CLSA data, and the approach we used to create the CII can be applied to other epidemiological studies that use neuropsychological batteries.

## Data Availability

These data are available publicly through an application to CLSA — https://www.clsa-elcv.ca/data-access. The CII is available as a derived variable or by contacting the first author for the SPSS syntax.

## Abbreviations

AF/AF2: Animal Fluency
CIHR: Canadian Institutes of Health Research
CII: Cognitive impairment indicator
CLSA: Canadian Longitudinal Study on Aging
CNS: Central nervous system
MAT: The Mental Alternation Test
OR: Odds ratio
REY I: Rey Auditory Verbal Learning Test immediate recall
REY II: Rey Auditory Verbal Learning Test 5-min delayed recall
TIA: Transient ischemic attack
TBI: Traumatic brain injury

## Footnotes

1 Although not described here, a CII based on three or more tests abnormally low as a cut off are available as a derived variable from CLSA for researchers who wish to use the stricter criterion.
